# Sunlight may increase the FDG uptake value in primary tumors of patients with non-small cell lung cancer

**DOI:** 10.3892/ol.2013.1112

**Published:** 2013-01-07

**Authors:** HASAN MUTLU, ABDULLAH BÜYÜKÇELIK, ESER KAYA, MUSTAFA KIBAR, ERTUĞRUL SEYREK, SINAN YAVUZ, ZÜLEYHA ÇALIKUŞU

**Affiliations:** 1 Department of Medical Oncology, Acibadem Kayseri Hospital, Kayseri 38000;; 2 Department of Internal Medicine, Acibadem University School of Medicine, Istanbul;; 3 Department of Nuclear Medicine, Acibadem Kayseri Hospital, Kayseri 38000;; 4 Department of Nuclear Medicine, Acibadem Adana Hospital, Adana, Turkey; 5 Department of Medical Oncology, Acibadem Adana Hospital, Adana, Turkey

**Keywords:** lung cancer, maximal standardized uptake value, season, positron emission tomography

## Abstract

Currently, positron emission tomography with computerized tomography (PET-CT) is the most sensitive technique for detecting extracranial metastases in non-small cell lung cancer (NSCLC). It has been reported that there is a correlation between the maximal standardized uptake value (SUV_max_) of primary tumors and prognosis in patients with NSCLC. The effect of sunlight exposure on PET-CT SUV_max_ value is not known. Therefore, we aimed to evaluate the effect of sunlight exposure on PET-CT SUV_max_ value in patients with NSCLC. A total of 290 patients with NSCLC from two different regions of Turkey (Kayseri, n=168 and Adana, n=122) that have different climate and sunlight exposure intensity, were included in the study. Age, gender, histology of cancer, cancer stage, smoking status, comorbidity and SUV_max_ of the primary tumor area at the time of staging were evaluated as prognostic factors. In the multivariate analysis, we detected that the region was the only independent factor affecting SUV_max_ (P=0.019). We identified that warmer climate and more sunlight exposure significantly increases the SUV_max_ value of the primary tumor area in patients with NSCLC. Further studies are warranted to clarify the issue.

## Introductıon

Lung cancer is one of most common types of cancer worldwide and it is the leading cause of cancer-related mortality in both males and females (1). Approximately 85% of lung cancers are non-small cell lung cancer (NSCLC). Squamous cell carcinoma, adenocarcinoma and large cell carcinoma are common histologies of NSCLC. The 5-year survival rates in patients with NSCLC vary with the stage of NSCLC. In tumor node metastasis (TNM) stage I, the 5-year survival rate is 73% (2), while in stage IV it is 13% (3). The 5-year survival rate of all patients with NSCLC is 15.7%. Cancer stage (4), performance status (5), ethnicity (6), histopathology (histological subtype, grade, lymphovascular invasion) (7–13), age (14,15), gender (16), carcinoembryonic antigen (CEA) and visceral pleural invasion (17) are prognostic factors in NSCLC. Currently, biomarkers such as p53 (18), K-ras (19), B cell lymphoma-2 (Bcl-2) (20), thyroid transcription factor 1 (TTF-1) (21), epidermal growth factor receptor (EGFR) (22), human epidermal growth factor receptor (HER-2 receptor) (23) and vascular endothelial growth factor (VEGF) (24) have been evaluated as new prognostic factors. Positron emission tomography (PET) with the glucose analogue, 2-[^18^F]-fluoro-2-deoxy-d-glucose (FDG) has been successfully used in various stages of care for patients with NSCLC, including staging procedures, radiotherapy planning and evaluation of the response to treatment (25). In addition, PET combined with computerized tomography (PET-CT) is also used for the evaluation of solitary pulmonary nodules (26). While PET-CT has a good negative predictive value in the evaluation of lymph nodes, it has a poor positive predictive value (27). At present, PET-CT is accepted as the most sensitive technique for detecting extracranial metastases from NSCLC (27,28). Since the introduction of PET-CT, a number of studies investigated whether maximal standardized uptake value (SUV_max_) is a prognostic and/or predictive parameter. Meta-analyses revealed that there is a correlation between SUV_max_ and prognosis in patients with NSCLC (25,29). These studies demonstrated that high SUV_max_ is associated with poor prognosis. Studies have shown that PET-CT has a predictive value in indicating the effectiveness of various types of chemotherapy (30,31).

Sufficient solar light exposure and vitamin D level may decrease the morbidity and mortality in patients with cancer. There is an inter-regional variability in solar light exposure in Turkey. The Mediterranean region has a warmer climate, whereas internal regions, including inner Anatolia are colder, particularly during winter. Therefore, annual solar light exposure is more intensive in the Mediterranean region than in internal regions (Table I). The effect of sunlight exposure on PET-CT SUV_max_ value is not known. Therefore, in the current study, we aimed to evaluate the effect of sunlight exposure on PET-CT SUV_max_ value in patients with NSCLC.

## Materials and methods

### Subjects

Patients with NSCLC from two different regions of Turkey (Kayseri and Adana, which have different climate and sunlight exposure intensity) were included in this study. Between 2009 and 2012, a total of 290 consecutive patients with NSCLC from Acibadem Kayseri Hospital and Acibadem Adana Hospital were analyzed retrospectively, using hospital records. All patients had a new diagnosis of NSCLC and PET-CT was used for baseline staging. The patients were divided into two groups, the Kayseri region, with a colder climate (n=168) and the Adana region, with a warmer climate (n=122). Staging was made according to the 7^th^ version of TNM lung cancer staging system. Age, gender, histological subtypes of cancer, cancer stage, smoking status (current or former smoker), comorbidity and SUV_max_ of the primary tumor area at the time of staging were recorded. This study was reviewed and approved by Ethics Committee of Erciyes University, Kayseri, Turkey. Written informed patient consent was obtained from all patients.

### PET-CT protocol

Patients were intravenously injected with ∼10 mCi (370 MBq) FDG. After 1 h rest in a silent room, the patients were imaged using an integrated PET-CT camera. The PET-CT scan was performed using a Siemens Biograph 6 PET-CT (LSO, 3D). The CT portion of the study was performed without intravenous contrast medium and used for defining anatomical marks and for attenuation correction of the PET reconstruction. The two centers used the same PET-CT protocols and the same PET-CT machine.

### Statistical analysis

Descriptive tests, Chi-square tests, independent samples tests and covariate analyses were performed using Statistical Package for the Social Sciences 16.0 (SPSS 16.0, SPSS Inc., Chicago, IL, USA). P<0.05 was considered to indicate a statistically significant difference.

## Results

Annual solar light exposure was more intensive in the Mediterranean region than the internal region. The hours of sunshine per day for the Kayseri and Adana regions are given in Table I.

Patient characteristics are given in Table II. The mean ages of patients from Kayseri and Adana were 62.1±9.2 and 61.7±10.5, respectively (P=0.733). The male to female ratio was similar in the two groups (P= 0.457). There was no significant difference in the histology of cancer (P=0.117), cancer stage (P=0.188), smoking status (P=0.334) and diabetes mellitus between the two groups (P=0.699). The mean SUV_max_ of patients from the Kayseri region was 13.1±6.4 and 14.6±5.8 for the Adana region (P=0.038). In order to identify which factors effect SUV_max_, we performed univariate analysis. The results of univariate analysis are presented in Table III. This analysis revealed that the region was the only independent factor affecting SUV_max_ (P=0.027).

## Discussion

In the present study, we investigated whether SUV_max_ is affected by sunlight exposure. We identified that SUV_max_ is significantly different in two different climates in Turkey. The subjects in the Adana region, which has a warmer climate and a greater annual average sunlight exposure, had a higher SUV_max_ compared with that in the subjects in the Kayseri region. The evaluated factors such as age, gender, histology, cancer stage, smoking and diabetes mellitus had no effect on SUV_max_. We identified that sunlight exposure was the only factor that significantly affected the SUV_max_ of the tumor area in patients with NSCLC. We suggest that higher SUV_max_ may be a prognostic factor for poor survival in lung cancer; however, our results oppose previous studies. In previous studies, patients who had more sunlight exposure or lived in a warmer climate had a better prognosis regarding survival. It was thought that this survival advantage may depend on vitamin D3 (1,25(OH)_2_D3) that is produced by ultraviolet B light isomerization of 7-dehydrocholestrol in the epidermis (32,33). Vitamin D3 exerts antiproliferative effects and inhibits growth and metastasis of lung cancer cells (34–40). A prospective study analyzed serum vitamin D levels in 6,937 patients with lung cancer (41). The association between the serum level of vitamin D3 and lung cancer risk was observed as the highest vs. lowest tertile with an odds ratio of 0.72. It has been demonstrated that improved survival in early stage lung cancer is associated with higher circulating levels of vitamin D3 (42,43). Patients diagnosed in summer and autumn are associated with improved survival compared to those diagnosed in winter. Cumulative sunlight exposure in the months preceding diagnosis is also a predictor of subsequent survival, although the season of diagnosis is a stronger predictor than cumulative sunlight exposure (44).

We identified that warmer climate and greater sunlight exposure significantly increases the SUV_max_ value in the primary tumor area in patients with NSCLC. Further studies are warranted to clarify the correlation between high SUV_max_, greater sunlight exposure, vitamin D3 and survival in patients with NSCLC.

## Figures and Tables

**Table I:** Hours of sunshine per day in the Kayseri and Adana regions of Turkey.

	Average hours of sunshine per day	
Months	Kayseri region	Adana region
January	3	5
February	4	5
March	5	6
April	6	7
May	8	10
June	10	11
July	12	11
August	11	11
September	9	9
October	7	7
November	5	6
December	3	5
Average (month)	210.9	237.9
Average (year)	2531	2855

P=0.475 between the two groups.

**Table II:** Patient’s baseline characteristics.

Parameters	Kayseri region	Adana region	P-value
Age (years)	62.1±9.2	61.7±10.5	0.733
Gender (n)			0.457
Male	158	112	
Female	10	10	
Histology			0.117
Squamous cell carcinoma	63	42	
Adenocarcinoma	49	33	
Others	1	6	
Not otherwise specified	55	41	
TNM stage			0.188
Stage 1	4	3	
Stage 2	17	23	
Stage 3	75	52	
Stage 4	72	44	
Smoking			0.334
Yes	129	101	
No	36	21	
Diabetes mellitus	8	7	0.699
SUV_max_ (mean ± SD)	13.1±6.4	14.6±5.8	0.038

SD, standard deviation; TNM, tumor node metastasis; SUV_max_, maximal standardized uptake value.

**Table III:** Results of univariate analysis between SUV_max_ and various parameters.

Parameter	P-value
Region	0.027
Age	0.103
Gender	0.168
Histology	0.906
Stage	0.901
Smoking status	0.668
Diabetes mellitus	0.142
